# Positive mental health, depression and burnout in healthcare workers during the second wave of COVID‐19 pandemic

**DOI:** 10.1111/jpm.13099

**Published:** 2024-08-19

**Authors:** Chiara Ruini, Giorgio Li Pira, Erika Cordella, Francesca Vescovelli

**Affiliations:** ^1^ Department for Life Quality Studies University of Bologna Rimini Italy; ^2^ Department of Psychology “Renzo Canestrari” University of Bologna Bologna Italy; ^3^ Azienda Universitaria Ospedaliera Policlinico di Sant'Orsola, IRCCS Bologna Italy; ^4^ Centro Regionale Trapianti Emilia Romagna (CRT ER) Bologna Italy

**Keywords:** burnout, COVID‐19 pandemic, depression, flourishing, health workers, mental health, resilience

## Abstract

**What is known on the subject?:**

- In the past 2 years, the COVID‐19 pandemic had a robust negative impact on the mental health of healthcare providers, with increasing rates of depression, anxiety, acute stress and burnout.
- Healthcare workers experiencing poor mental health are reluctant to seek help and treatment because they are afraid of being stigmatized and excluded by their colleagues and employers.
- During the pandemic positive emotions, resilience and psychological well‐being buffered distress and burnout in healthcare workers.

**What this paper adds to existing knowledge?:**

- This paper describes positive mental health, depression, anxiety and burnout in healthcare workers during the second wave of the pandemic.
- Forty‐eight per cent of healthcare workers were flourishing (high levels of positive emotions and well‐being), 10% languishing (absence of well‐being and positivity).
- Flourishing individuals reported lower levels of depression, anxiety and burnout.
- These findings documented a relevant number of resilient healthcare workers, who restored/maintained their well‐being also under stressful conditions.
- Vulnerable healthcare workers were less than 20%, and they reported severe anxiety, depression and burnout.
- No differences emerged between languishing and moderate mental health groups in their levels of anxiety, depression and burnout.

**What are the implications for practice?:**

- The condition of flourishing is the only one that provides protection from depression and anxiety and burnout, while moderate mental health does not differ substantially from the languishing state.
- The study confirms the importance of maintaining and/or promoting the well‐being of healthcare workers.
- Interventions for promoting positive mental health of vulnerable workers are needed.
- Mental health nurses can have the skills and expertise for evaluating early symptoms of psychological distress and for implementing interventions for promoting and restoring well‐being.
- These interventions may include informational campaign (i.e. preparing and distributing pamphlets and guidelines) and emotional support programmes (psychoeducation and training, mental health support team, peer support and counselling) that can be delivered also via digital platforms.

**Abstract:**

**Introduction:**

Few studies focused on healthcare workers' positive mental health (i.e. high levels of psychological well‐being) and its association with anxiety, depression and burnout in the second wave of the pandemic.

**Aims:**

To evaluate the protective role of well‐being in buffering burnout and psychological distress.

**Methods:**

We evaluated 173 Italian healthcare workers with indicators of psychological distress (Depression Anxiety Stress Scales [DASS]‐21), burnout (Copenhagen Burnout Inventory [CBI]) and positive mental health (Mental Health Continuum) and we cross‐classified them according to their levels of mental health (flourishing, languishing and moderate mental health) and their levels of anxiety, depression and burnout.

**Results:**

Forty‐eight per cent of health workers were classified as flourishing, 10% as languishing and 42% as moderate mental health. Flourishing individuals presented lower scores on DASS and CBI scales, whereas no differences emerged between languishing and moderate mental health groups. More than 80% of health workers with clinically significant symptoms of anxiety, depression and risk of burnout were classified as not flourishing.

**Discussion:**

This investigation documented the presence of flourishing mental health in almost half of the sample of Italian healthcare workers. However, those with moderate or languishing mental health manifested higher levels of anxiety, depression and higher risks of burnout.

**Implication for Practice:**

The study confirms the importance of maintaining and/or promoting the well‐being of this population, with a crucial role of mental health nurses who can easily approach other healthcare workers and provide them informational (training, guidelines) and emotional support programmes (psychoeducation, mental health support team, peer support and counselling) when facing adverse working conditions.

## 1 INTRODUCTION

From 2020 to 2022, the COVID‐19 pandemic had a robust negative impact on the mental health of the global populations, with increasing rates of depression, anxiety, acute stress and post‐traumatic stress disorders (Akay, 2022; Crocker et al., 2022). Among different subgroups, nurses and healthcare providers have been particularly investigated, in the light of their work in direct contact with patients infected with the COVID‐19 virus (Hurst et al., 2022; Liang et al., 2022; Sani et al., 2022; Veitch & Richardson, 2021; Zhang et al., 2021). They have been considered ‘heroes’ across different nations, especially during the first wave of pandemic (Brennan, 2020; Cox, 2020; Mascayano et al., 2022). However, various investigations assessed their mental health and found that these ‘heroes’ manifested symptoms such as traumatic stress, depression, anxiety, psychological (and physical) exhaustion, especially for frontline workers (Elgohary et al., 2021; Hurst et al., 2022; Labrague et al., 2021; Mascayano et al., 2022; Narita et al., 2023; Sani et al., 2022; Zhang et al., 2021). During the first wave of pandemic (when the COVID‐19 vaccines were not available and face masks and other personal protective equipment were scarce), health workers felt particularly vulnerable since they had to deal with an increased workload due to the massive contagion, and with an increased mortality rate among their patients (Crocker et al., 2022; Labrague et al., 2021; Pang et al., 2021; Sani et al., 2022; Webb, 2021).

Various studies (Butler, 2022; Carmassi et al., 2021; Crocker et al., 2022; Mendez‐Pinto et al., 2023) investigated the prevalence of depression in healthcare workers during the pandemic and found that major depressive disorder was common, particularly in female workers. Among female workers, those who worked as frontline nurses reported that the risk of transmitting the infection to their household members, and the fear of death were the factors that significantly affected their levels of depression, anxiety and stress (Maideen et al., 2022; Mendez‐Pinto et al., 2023; Pang et al., 2021).

Furthermore, across nations, a high percentage of health workers reported personal and professional burnout and work stress (Merino‐Godoy et al., 2022; O'Higgins et al., 2022; Wilson et al., 2022; Zhang et al., 2021), that were significantly associated with other psychopathological symptoms, as post‐traumatic stress depression and anxiety symptoms (Mendez‐Pinto et al., 2023; Narita et al., 2023; Pang et al., 2021; Webb, 2021). However, the pandemic has only exacerbated work stress and burnout, which have emerged as chronic problems in healthcare settings, with long‐term negative consequences (Crocker et al., 2022; Wilson et al., 2022).

Moreover, it has been previously observed that healthcare workers experiencing poor mental health are reluctant to seek help and treatment because they are afraid of being stigmatized and excluded by their colleagues and employers (Butler, 2022). Thus, depression, anxiety and burnout easily became chronic and vexing conditions for this populations.

Besides the assessment of risk factors for poor mental health during the pandemic, some investigations also explored healthcare workers' protective factors, such as coping strategies, resilience and positive mental health. Various investigations reported the use of positive coping strategies in healthcare workers during the pandemic, such as self‐care, family and social support (Maideen et al., 2022; Sani et al., 2022). Resilience was found to be a mediator for nurses' mental health during the first wave of pandemic (Labrague et al., 2021) and it was found to influence burnout via the mediation of positive and negative emotions in a sample of healthcare workers in Whuan (Zhang et al., 2021).

Bassi et al. (2021) explored positive mental health in a group of Italian health workers during the first wave of pandemic and found that those having higher levels of well‐being reported lower post‐traumatic stress symptoms. However, only a small proportion of them (33%) manifested flourishing mental health. According to the dual continuum model (Keyes, 2002), flourishing mental health can be diagnosed when individuals experience high levels of hedonic well‐being (positive affect and pleasure) and high levels of psychological and social well‐being. Conversely, the condition of languishing has been described as a state in which an individual is devoid of positive emotions towards life, and is not functioning well either psychologically or socially, without fulfilling the criteria for a DSM disorder (Keyes et al., 2012).

Flourishing mental health was shown to predict mental illness in the general population (e.g. the risk of developing anxiety and depressive disorders), to influence recovery from an affective disorder (Iasiello et al., 2019; Keyes et al., 2010; Vescovelli et al., 2020) and to provide a buffering effect when facing chronic and acute stress (Westerhof & Keyes, 2010). In a pre‐pandemic investigation with North American medical students, flourishing mental health was found to attenuate some adverse consequences of burnout, such as unprofessional behaviours or falsification of medical reports (Dyrbye et al., 2012). It was also documented that mental health predictors paralleled those of burnout (i.e. year of specialty; ethnicity, gender) (Morgan et al., 2020) and that burnout was inversely correlated to positive emotions (Zhang et al., 2021).

With few exceptions (Anozie et al., 2020; Bassi et al., 2021; Dyrbye et al., 2012; Morgan et al., 2020), no other investigations explored the mental health condition of healthcare workers using this dual continuum model, where indicators of psychopathology were evaluated with indicators of positive functioning. Furthermore, most of data on healthcare workers' mental health were collected before or during the acute phase of the pandemic, while the subsequent phases received less attention.

The aim of this investigation was to evaluate positive mental health (flourishing) in a sample of Italian healthcare workers during the second wave of the pandemic, when vaccines were just released, and personal protective equipment were largely available. In that phase of the pandemic, the COVID‐19‐emergency working conditions began to fade away, while chronic stressors and heavy workload persisted in healthcare settings. The purpose of the present investigation was to extend prior research by evaluating the role of positive mental health in protecting healthcare workers from depression, anxiety and burnout. The results of this investigations could imply an important role for mental health nurses in light of their expertise in assessing these symptoms and in implementing evidence‐based interventions for promoting positive mental health in healthcare systems.

## 2 METHODS

### 2.1 Sample

Participants were recruited from several hospitals and nursing homes in Northern Italy by contacting their administrative officers/CEO, Directors. Local nurses, physicians and psychologists' professional associations were also contacted by the researchers. They received a letter with a description of the study aims and a request for participation. After the institutions' Ethical Board Commissions provided approval for the study and accepter their voluntarily participations, a web link with an online survey was sent and distributed among healthcare workers. As additional recruitment, consenting participants were asked to distribute the web link to other colleagues in their healthcare settings. The online survey contained questions about sociodemographic characteristics, work conditions and information about COVID‐19 infection. Healthcare workers could access the survey only after signing the informed consent form. Anonymity was preserved through assignment of an alpha numeric code. Median survey completion time was 25 min. Data collection took place between March 2021 and January 2022.

### 2.2 Assessment

Sociodemographic data collection included age, gender, marital status and education. Work‐related data were gathered on profession, job seniority, workplace and direct contact with patients during the pandemic. Information was collected also regarding healthcare infection with COVID‐19 virus (yes/no).

*Positive mental health* was assessed using the following questionnaire:

*Mental Health Continuum Short‐Form* (MHC‐SF; Keyes, 2005; Petrillo et al., 2015): It consists of 14 items that measured the frequency of 3 well‐being domains: 3 items for emotional well‐being (EWB), 6 items for psychological well‐being (PWB) and 5 items for social well‐being (SWB) according to a Likert scale ranging from 0 ‘never’ to 5 ‘everyday’. A total well‐being score was calculated with a Cronbach'*α* value of 0.950 in this sample. According to Keyes's model (2002), three categorical mental health diagnoses can be computed: (a) *flourishing*: when participants experienced at least 1 of the 3 EWB symptoms and 6 of the 11 PWB/SWB symptoms ‘every day’ or ‘almost every day’ in the past month; (b) *languishing*: when participants experienced at least 1 of the 3 EWB symptoms and 6 of the 11 PWB/SWB symptoms ‘never’ or ‘once or twice’ during the past month; (c) *moderate mental health*: when participants were neither flourishing nor languishing.

*Psychological distress* was assessed using the following questionnaire:

*Depression Anxiety Stress Scales‐Short Version* (DASS‐21; Lovibond & Lovibond, 1995): It consists of 21 items (7 items per subscale: depression, anxiety and stress). Participants were asked to score every item on a Likert scale from 0 (did not apply to me at all) to 3 (applied to me very much). Sum scores were computed by adding up the scores on the items per (sub)scale. Sum scores for the total DASS‐total scale thus range between 0 and 63, and those for each of the subscales may range between 0 and 21. The reliability of the DASS‐21 (total score) in this study population was α 0.947. According to Lovibond and Lovibond (1995), specific subscale cut off scores for identifying severity levels of depression, anxiety and stress can be used to classify participants from normal‐mild symptoms to extremely severe. In this research, health workers were categorized according to: (1) their scores on the DASS depression subscale (low = absence to moderate depression when scores ranged from 0 to 10; high = severe to extremely severe depression when scores were ≥11); (2) their scores on the DASS anxiety subscale (low = absence to moderate anxiety when scores ranged from 0 to 7; high = severe to extremely severe anxiety when scores were ≥8).

*Copenhagen burnout inventory* (CBI, Kristensen et al., 2005): It is a tool for measuring burnout and it includes three different subscales: (a) personal burnout, (b) work‐related burnout and (c) client/user‐related burnout. It consists of 19 items with response on a 5‐point Likert scale based on the frequency with which the individual experiences the situation described. According to Fiorilli (2015), specific cut‐off‐score can be used to identify individuals at risk for developing burnout. In this investigation, we categorize individuals according to their total CBI score: −low = low to medium risk (scores up to 50); −high = moderate to high risk (score ≥51). The Cronbach α in this sample was 0.90.

### 2.3 Statistical analyses

Skewness and kurtosis values were calculated and indicated that the data satisfied the normality assumption for all the measures used. Descriptive statistics were used to describe the final sample of Italian healthcare workers (mean/frequencies). Participants were then classified according to their positive mental health diagnosis (flourishing, moderate, languishing mental health) and frequencies were computed.

Comparisons among the three mental health categories were calculated using the multivariate analysis of variance (MANOVA), with the mental health category as a fixed factor and CBI subscales and DASS subscales as dependent variables, respectively. Univariate tests were subsequently calculated for the single dependent variables and Bonferroni post hoc tests were used. The significance level was set at *p* < .05. The partial eta squared as a measure of effect size was calculated considering a value of 0.1 as a large effect, a value of 0.04 as a medium effect and a value of 0.01 as a small effect (Huberty, 2002). The significance level was set at *p* < .05.

Then, healthcare workers were categorized according to their levels of depression, anxiety (DASS scores; 0 = mild to moderate; 1 = severe) and risk of burnout (CBI scores; 0 = low risk; 1 = high risk) and subsequently we cross‐classified them according to their levels of depression, anxiety and burnout and their mental health categories. Considering that languishing individuals were expected to be around 10% of the sample, and that cross‐tabulation statistics require a minimum number of cases for each cell, we have aggregated the categories of moderate mental health and languishing. Thus, the total sample was categorized into ‘flourishing’ or ‘not flourishing’ healthcare workers. We calculated Chi‐square values. In order to control for the many pairwise comparisons and to prevent alpha inflation, Bonferroni correction was applied and the alpha value of 0.05 was divided by the total number of comparisons made. Accordingly, the *p* value was set at .008.

Analyses were performed with the Statistical Package for the Social Sciences, version 28.

## 3 RESULTS

A total of 173 healthcare workers completed the survey. After data cleaning, 5 participants were excluded due to missing data (one or more questionnaires not answered). The final dataset included 168 responders: *n* = 137 female (81.5%); *n* = 31 males (18.5%). Their mean age was 43.68 years (SD = 10.741; age range: 22–71). Based on the Italian classification system of health professions, participants were physicians (8.7%), nurses and midwives (23.7%), nurses working in ageing facilities (19.1%), psychologists (6.9%), social workers, professionals in technical (e.g. technicians in radiology, biomedical lab; 2.9%) and rehabilitation areas (e.g. physiotherapists), and healthcare assistants (25.4%). They were divided into two categories based on their workplace: the category ‘*frontline workers*’ included healthcare workers working directly in contact with hospitalized patients or in nursing facilities, or with outpatients, but with a physical contact with them. The category ‘*second‐line workers*’ comprised healthcare workers working in healthcare facilities, but without a direct/in person daily contact with patients (technicians in radiology, biomedical lab, administrative staff, etc.).

Regarding the three categories of the Mental Health Continuum, we found that 48% of the participants were in the flourishing category, 42% were in the moderate mental health category and 10% were in the languishing category. Their sociodemographic characteristics and working conditions are described in Table 1.

**TABLE 1:** Sociodemographic characteristics of the total and of the three MHC categories.

	Total sample (*n* = 168)	Flourishing (*n* = 82) M (SD)	Moderate (*n* = 68) M (SD)	Languishing (*n* = 18) M (SD)	*F*
Age	43.6 (10.74)	45.1 (10.1)	41.4 (11.3)	45.7 (10)	2.633
	**Total sample (*n* = 168)**	**Flourishing (*n* = 82; 48%) *n* (%)**	**Moderate (*n* = 68; 42%) *n* (%)**	**Languishing (*n* = 18; 10%) *n* (%)**	**Chi‐square**
Gender					
Male Female	31 (18.5%) 137 (81.5%)	15 (18.3%) 67 (81.7%)	15 (22.1%) 53 (77.9%)	1 (5.6%) 17 (94.4%)	2.579
COVID					
Yes No	24 (14.3%) 144 (85.7%)	14 (17.1%) 68 (82.9%)	7 (10.3%) 61 (89.7%)	3 (16.7%) 15 (83.3%)	1.488
Frontline workers					
Yes No	117 (69.6%) 51 (30.4%)	53 (64.6%) 29 (35.4%)	49 (72.1%) 19 (27.9%)	15 (83.3%) 3 (16.7%)	2.757
Years of work					
1 year More than 1 year	36 (21.4%) 132 (78.6%)	15 (18.3%) 67 (81.7%)	20 (29.4%) 48 (70.6%)	1 (5.6%) 17 (94.4%)	5.747

A MANOVA was performed to compare the three MHC categories according to their DASS scores and CBI scores. Simple main effects analysis showed that belonging to a certain category of MHC did have a statistically significant effect on all the DASS‐21 subscales: respectively on depression score (*f*_2,165_ = 18.414; *p* ≤ .001, partial eta squared = 0.182), on anxiety score (*f*_2,165_ = 7.131; *p* ≤ .001, partial eta squared = 0.080) and on stress score (*f*_2,165_ = 11.479; *p* ≤ .001, partial eta squared = 0.122). Univariate tests and post hoc comparison showed that the flourishing healthcare workers had lower score on all the subscales (*p* < .05) compared to the languishing and moderate groups, except for the stress subscale, where the flourishing and languishing groups did not significantly differ (*p* = .377).

Simple main effects analysis showed that belonging to a certain category of MHC did have a statistically significant effect also on CBI subscales: respectively on personal burnout (f _2165_ = 14.004; *p* ≤ .001, partial eta squared = 0.145) client burnout (f _2165_ = 4.5; *p* ≤ .05, partial eta squared = 0.052) and work burnout (f _2165_ = 15.546; *p* ≤ .001, partial eta squared = 0.159). Univariate tests and post hoc comparison showed that the flourishing healthcare workers had lower scores on all the subscales (*p* < .05) compared to the languishing and moderate groups, except for the client burnout subscale, where the flourishing and languishing groups did not significantly differ (*p* = .864) (Table 2).

**TABLE 2:** Differences of the mean score on depression, anxiety and risk of burnout among the three MHC categories.

	Total sample (*n* = 168) M (SD)	Flourishing (*n* = 82) M(SD)	Moderate (*n* = 68) M (SD)	Languishing (*n* = 18) M(SD)	*F*
DASS Depressio	3.8 (3.9)	2.1 (2.5)^a^	5.5 (4.7)^b^	5.4 (2.9)^b^	18.414**
DASS stress	6.5 (4.4)	4.9 (4.0)^a^	8.3 (4.6)^b^	6.7 (3.4)^a,b^	11.479**
DASS anxiety	2.9 (3.2)	2.0 (2.2)^a^	3.7 (3.9)^b^	4.4 (3.3)^b^	7.131**
DASS total	13.3 (10.15)	9.1 (7.8)a	17.4 (11.9)^b^	16.5 (7.6)^b^	14.713**
CBI work	34.3 (21.9)	25.5 (19.2)a	43.4 (19.9)^b^	40.5 (25.2)^b^	15.546**
CBI client	21.1 (22.0)	31.7 (18.6)^a^	26.9 (24.8)^b^	20.8 (22.7)^a^	4.500*
CBI personal	39.8 (21.1)	16.3 (18.1)^a^	48.2 (19.7)^b^	45.4 (23.3)^b^	14.004**
CBI total	32.2 (18.8)	25.5 (16)^a^	40.4 (17.7)^b^	36.4 (20.7)^b^	16.221**

*Note*: Subscripts a and b show graphically the results of the Bonferroni post‐hoc test. Mean is significantly different from another mean if they do not share the same letter.

* *p* < .05.

** *p* < .001.

Regarding the categories of depression, anxiety and burnout in the total sample of healthcare workers, we found only a minority of participants with high scores in depression (7.1%), in anxiety (11.3%) and with high risk of burnout (17.3%) (see Table 3). The chi‐square tests were used to assess the relationship between the categories of flourishing and ‘not flourishing’ and the levels of depression (low score = absence to moderate; or high score = severe to extremely severe); of anxiety (low score = absence to moderate; or high score = severe to extremely severe) and the risk of burnout (low = low to medium; high = moderate to high), respectively. Chi squared values were all significant: we found a significant association between mental health categories and depression (*χ* ^2^_(1,168)_ = 8.474, *p* < .008), anxiety (*χ* ^2^_(1,168)_ = 12.566, *p* < .001) and burnout risk (*χ* ^2^_(1,168)_ = 8.539, *p* < .008). In particular, 91% of individuals with severe depression, 89% of individuals with severe anxiety and 75% of individuals with high risk of burnout were categorized as ‘not flourishing’.

**TABLE 3:** Differences in frequency distribution of depression, anxiety and risk of burnout between flourishing versus not flourishing Healthcare Workers.

	Total sample (*n* = 168)	Flourishing (*n* = 82) *n* (%)	Not flourishing (*n* = 86) *n* (%)	Chi‐square
Depression				
Low score High score	156 (92.9%) 12 (7.1%)	81 (98.8%) 1(1.2%)	75 (87.2%) 11 (12.8%)	8.474**
Anxiety				
Low score High score	149 (88.7%) 19 (11.3%)	80 (97.6%) 2 (2.4%)	69 (80.2%) 17 (19.8%)	12.566**
Burnout risk				
Low score High score	139 (82.7%) 29 (17.3%)	75 (91.5%) 7 (8.5%)	64 (74.4%) 22 (25.6%)	8.539**

** *p* < .008.

## 4 DISCUSSION

The aim of this investigation was to evaluate positive mental health in a sample of Italian healthcare workers, after the acute phase of the COVID‐19 pandemic and to verify if the presence of well‐being might protect healthcare workers from depression, anxiety and risk of burnout. These conditions have been considered frequent and chronic problems in healthcare settings, that the pandemic has tremendously exacerbated (Akay, 2022; Crocker et al., 2022; Merino‐Godoy et al., 2022; Pang et al., 2021; Veitch & Richardson, 2021; Webb, 2021). The first important finding of the present research is that almost half of our sample of healthcare workers (48%) were diagnosed as flourishing (i.e. they reported high levels of hedonic well‐being and positive functioning in life), while only a minority (10%) were in the languishing condition (see Table 1). These data provide a novel, more positive picture of Italian health workers compared to the results emerged in recent publication (Bassi et al., 2021), where flourishing health workers were only 28% of the sample. Our data are more in line with those reported by Anozie et al. (2020), who documented 47% of flourishing individuals in a sample of African healthcare workers, while languishing individuals were almost 25% of their sample. This could be explained by the fact that the pandemic had a less severe impact on African countries, where hospitals were less stressed by COVID‐19 cases, because of the youthfulness of the population and its lower density (Vallée, 2023). In fact, the previous Italian investigation (Bassi et al., 2021), was conducted during the first wave of COVID‐19 pandemic, when healthcare workers were under severe conditions of stress and vulnerability because facial masks, protective devices and COVID‐19 vaccines were not available. Conversely, our results pertain to the actual state of healthcare workers, when the pandemic has become a chronic condition, without traumatic, life‐threatening aspects (Bassi et al., 2021). Another investigation conducted in the Italian population prior to the pandemic found that 28.5% of the adult sample was flourishing and 10.5% was languishing (Petrillo et al., 2015). Thus, as reported by other epidemiological studies (Capone et al., 2020; Keyes, 2006; Westerhof & Keyes, 2010) the condition of languishing is usually reported by a small proportion of individuals (around 10%), flourishing around one third of the samples, while the most frequent condition is moderate mental health. These frequencies provide important clinical data, since the presence/absence of well‐being was found to predict mood and anxiety disorders in several longitudinal investigations (Iasiello et al., 2019; Schotanus‐Dijkstra et al., 2019; Westerhof & Keyes, 2010; Wood & Joseph, 2010). Forty‐eight per cent of our sample of healthcare workers reported high levels of well‐being and positive functioning after the traumatic experience of the first acute phase of the pandemic. Thus, these findings depict a relevant number of resilient healthcare workers, who restored/maintained their well‐being also under stressful conditions, as already documented in previous investigations (Labrague et al., 2021; Merino‐Godoy et al., 2022; Toh et al., 2021; Zhang et al., 2021).

In fact, flourishing healthcare workers also reported lower levels of depression, anxiety, stress and burnout compared to the other two mental health groups as documented by the MANOVA. Interestingly, at post hoc analyses, individuals in the moderate mental health group did not differ significantly from those in the languishing condition (see Table 2). This is a novel, important finding of the present investigation, since previous research has documented that either the three mental health groups differed in terms of mental illnesses and stress (Capone et al., 2020) or that the languishing group was at higher risk of mental illness (Bassi et al., 2021; Iasiello et al., 2019).

The cross classification of the sample according to the categories of flourishing versus ‘not flourishing’ confirmed these results (see Table 3). For example, of the 82 flourishing individuals in our sample only 1 belonged to the group reporting high scores on the DASS depression subscale, 2 individuals belonged to the group reporting high scores on the DASS anxiety subscale and 7 flourishing individuals belonged to the group with high risk of burnout. Conversely, 11 out of 12 healthcare workers with severe or extremely severe depression were categorized in the ‘not flourishing’ group. Similar data were obtained for participants reporting severe anxiety symptoms (17 out of 19 in not flourishing group) and higher risk of burnout (see Table 3). In this case, only 8.5% of individuals in the flourishing group documented high scores of burnout.

Other authors in previous investigations decided to dichotomize mental health categories into flourishing or not flourishing group (Doré et al., 2020; Schotanus‐Dijkstra et al., 2019) and found similar results: individuals in the ‘not flourishing mental health’ condition had higher risks of manifesting high levels of anxiety and depressive symptoms (Doré et al., 2020), and a poorer recovery from an affective disorder (Iasiello et al., 2019; Schotanus‐Dijkstra et al., 2019) compared to those in the flourishing group. The present investigation confirms the conclusions of these previous research: the condition of flourishing is the only one that provides protection from common mental health problems (i.e. depressive and anxious symptoms), while moderate mental health (the most frequent condition in all studies) does not differ substantially from the languishing state.

The same considerations can be drawn for the risk of burnout: in our sample of health workers, 29 of them reported high levels of burnout, and most of them were categorized in the ‘not flourishing’ group. Previous investigations in healthcare workers documented the strong correlations between burnout, depressive and anxiety disorders (Barello et al., 2020; Ghio et al., 2021; Merino‐Godoy et al., 2022; Wang et al., 2023; Wilson et al., 2022), with concomitant manifestations of insomnia, fatigue and post‐traumatic stress symptomatology. At the same time, a recent investigation documented a robust, inverse relationship among burnout, purpose in life and personal fulfilment (two core dimensions of flourishing) in healthcare workers (O'Higgins et al., 2022). In this case, higher levels of purpose were related to increased levels of personal accomplishment, a sense of professional identity and job satisfaction that positively influenced healthcare workers' levels of burnout during the pandemic. Even though we did not assess purpose in life in details, the MHC questionnaire contains items for measuring the sense of personal and societal growth and meaning in life. Thus, our data confirm this recent research and sustain the role of personal growth and purpose in protecting healthcare workers from burnout, particularly under the stressful experience of COVID‐19 pandemic.

## 5 LIMITATIONS

This study is limited by its preliminary nature, the self‐selected, homogenous sample and the use of self‐reports as assessment methodology. This fact leaves the possibility of self‐report bias to inflate some of the significant correlations and it limits the external validity of the findings. Furthermore, even though we have differentiated frontline and second‐line healthcare workers, we did not provide additional information on specific specialist areas (i.e. respiratory acute wards or intensive care wards) where the pandemic could have had a more detrimental effect on clinicians' mental health.

Finally, the cross‐sectional study design precluded conclusions about causality among variables. Follow‐up and longitudinal studies should be performed to monitor positive mental health over the chronic course of the COVID‐19 pandemic and its relationships with psychopathology and burnout in healthcare workers.

## 6 CONCLUSIONS

To the best of our knowledge, this is the first investigation during the second wave of the COVID‐19 pandemic that was specifically focused on health workers' mental health and its correlates in terms psychopathology and burnout. We found that almost 48% of our sample maintained their well‐being after the acute phase of the pandemic and only small proportions of them reported high levels of depression, anxiety and burnout. These individuals can be considered as more vulnerable (Veitch & Richardson, 2021; Webb, 2021), and they were mostly categorized under the ‘not flourishing’ group.

Considering that the pandemic has revealed the crucial role of health professionals in communities and societies around the world, this investigation provides confirmation to the importance of maintaining and/or promoting the well‐being of this population, particularly during health crises or under stressful working conditions (Buselli et al., 2021; Gray et al., 2021; Veitch & Richardson, 2021). A suitable model for addressing this issue was proposed by a group of European researchers (Tomlin et al., 2020). This phased model of mental health burden and responses suggests that different strategies to support clinicians' work and to prevent burnout could be applied according to the phase of crisis faced by the specific healthcare organization. Even though the model was developed during the COVID‐19 pandemic, it could be easily applied to other sources of psychosocial stress in the healthcare setting.

## 7 IMPLICATIONS FOR CLINICAL PRACTICE

Even if psychological interventions to support healthcare workers' mental health have been implemented during the pandemic, only few countries have published specific protocols, and none of them was specifically tailored to the promotion of resilience and psychological well‐being (Buselli et al., 2021; Wang et al., 2023). Nowadays, various effective interventions to promote well‐being are available, ranging from meditation techniques, to yoga, lifestyle modification and positive psychotherapies (van Agteren et al., 2021). These interventions have been found to be feasible, cost‐effective and deliverable in various healthcare settings.

Unfortunately, healthcare workers are often hesitant to seek treatment for their poor mental health, because of stigmatization and negative stereotypes about psychiatric disorders in healthcare organizations (Butler, 2022). Mental health nurses or those working in mental health settings can have a crucial role as peer experts in evaluating symptoms of depression, anxiety, stress and burnout observed and reported by other healthcare workers. In fact, they possess the expertise in common mental health problems, they have specific training for active listening and for building a therapeutic relationship and they share the same (or similar) occupational conditions of those who are experiencing these type of problems (Sampaio et al., 2015). Moreover, previous investigations published in this journal have documented that mental health nurses can be actively involved in co‐designing more inclusive workplaces (Stab & Hacker, 2020) or in realizing multidisciplinary support groups for healthcare workers, that buffered distress and excessive workload during the acute phase of the pandemic (Veitch & Richardson, 2021). Another review article summarized the various mental health interventions delivered to healthcare workers during recent infectious disease outbreaks (Zace et al., 2021): most of those interventions included informational (training, guidelines, prevention programmes) and emotional support programmes (psychoeducation and training, mental health support team, peer support and counselling), which were provided (or coordinated) by mental health nursers. For example, during the SARS pandemic the management of a hospital in Canada asked healthcare workers within the mental health settings (nurses and doctors) to prepare a pamphlet identifying signs of anxiety and stress and information about support resources, which was distributed across hospital's units. Furthermore, mental health nurses activated a confidential telephone support line for all hospital staff and it was used effectively by those in quarantine (Maunder et al., 2003). More recently, Gonzales et al. (2020) reported the experience of another hospital during the COVID‐19 pandemic where mental health nurses were strategically charged with taking personal protective equipment orders throughout the institution. This assignment gave them the opportunity to offer in‐person support to healthcare staff, including meditations, empathic listening, encouragement and support resource flyers. In other experiences, mental health nurses were involved in delivering psychoeducation about stress, anxiety and depressive symptoms through digital platforms or via video conference (Zace et al., 2021). Thus, mental health nurses or coaches are in the ideal position for facilitating the implementation of interventions for promoting and restoring well‐being in vulnerable healthcare workers.

If future longitudinal research would replicate the findings of this study, these interventions may become essential tools to be implemented in healthcare settings in order to prevent burnout and to foster health workers' positive mental health.

## 8 RELEVANCE STATEMENT

From 2020 to 2022, the COVID‐19 pandemic had a robust negative impact on the mental health of healthcare providers, with increasing rates of depression, anxiety, acute stress and burnout. In this investigation, we found that these conditions persisted also in the second wave of the pandemic, in almost 10–20% of the sample. However, 48% of healthcare workers were able to restore their well‐being, and were classified with flourishing mental health. Specific subgroups of healthcare workers present vulnerabilities in their mental health and mental health nurses have the skills and expertise for evaluating early symptoms of psychological distress and for implementing interventions for promoting and restoring well‐being. These interventions may include informational campaign (i.e. preparing and distributing pamphlets and guidelines) and emotional support programmes (psychoeducation and training, mental health support team, peer support and counselling) that can be delivered also via digital platforms. Mental health nurses may also be actively involved in co‐designing more inclusive healthcare workplaces to address mental health stigma often experienced by vulnerable healthcare workers.

## AUTHOR CONTRIBUTIONS

All authors significantly and equally contributed to the manuscript. CR and EC supervised the research and contributed to the study design and writing of the article. GLP and FV contributed to data collection data analyses and writing of the manuscript.

## ETHICS STATEMENT

The study conforms to the Declaration of Helsinki standards and was approved by the ethical boards of the hospitals/clinical institutions enrolled for the study implementation and data collection.

## Data Availability

Data available upon request to the authors.

## References

[jpm13099-bib-0001] Akay, A. (2022). The local and global mental health effects of the Covid‐19 pandemic. Economics and Human Biology, 45, 101095. 10.1016/j.ehb.2021.101095 35092869 PMC8750697

[jpm13099-bib-0002] Anozie, O. B. , Nwafor, J. I. , Nwokporo, E. I. , Esike, C. U. , Ewah, R. L. , Eze, J. N. , Azuogu, B. N. , & Ukaegbe, C. I. (2020). Mental health impact of COVID‐19 pandemic on health care workers in Ebonyi State, southeast, Nigeria. International Journal of Innovative Research in Medical Science, 5(9), 400–406. 10.23958/ijirms/vol05-i09/955

[jpm13099-bib-0003] Barello, S. , Palamenghi, L. , & Graffigna, G. (2020). Burnout and somatic symptoms among frontline healthcare professionals at the peak of the Italian COVID‐19 pandemic. Psychiatry Research, 290, 113129. 10.1016/j.psychres.2020.113129 32485487 PMC7255285

[jpm13099-bib-0004] Bassi, M. , Negri, L. , Delle Fave, A. , & Accardi, R. (2021). The relationship between post‐traumatic stress and positive mental health symptoms among health workers during COVID‐19 pandemic in Lombardy, Italy. Journal of Affective Disorders, 280, 1–6. 10.1016/j.jad.2020.11.065 PMC9188831 33220632

[jpm13099-bib-0005] Brennan, J. (2020). Heroes in healthcare; what's wrong with that? International Journal for Quality in Health Care, 32(9), 567–568. 10.1093/intqhc/mzaa100 32991691

[jpm13099-bib-0006] Buselli, R. , Corsi, M. , Veltri, A. , Baldanzi, S. , Chiumiento, M. , Lupo, E. D. , Marino, R. , Necciari, G. , Caldi, F. , Foddis, R. , Guglielmi, G. , & Cristaudo, A. (2021). Mental health of health care workers (HCWs): A review of organizational interventions put in place by local institutions to cope with new psychosocial challenges resulting from COVID‐19. Psychiatry Research, 299, 113847. 10.1016/j.psychres.2021.113847 33721785 PMC7920813

[jpm13099-bib-0007] Butler, R. M. (2022). Serious mental illness in healthcare and academia: A lived experience. Journal of Psychiatric and Mental Health Nursing, 29(5), 624–629. 10.1111/jpm.12862 35876216

[jpm13099-bib-0008] Capone, V. , Caso, D. , Donizzetti, A. R. , & Procentese, F. (2020). University student mentalwell‐being during COVID‐19 outbreak: What are the relationships between information seeking, perceived risk and personal resources related to the academic context? Sustainability, 12(17), 7039. 10.3390/su12177039

[jpm13099-bib-0009] Carmassi, C. , Cerveri, G. , Bertelloni, C. A. , Marasco, M. , Dell'Oste, V. , Massimetti, E. , Gesi, C. , & Dell'Osso, L. (2021). Mental health of frontline help‐seeking healthcare workers during the COVID‐19 outbreak in the first affected hospital in Lombardy, Italy. Psychiatry Research, 298, 113763. 10.1016/j.psychres.2021.113763 33545425 PMC7835096

[jpm13099-bib-0010] Cox, C. L. (2020). ‘Healthcare heroes’: Problems with media focus on heroism from healthcare workers during the COVID‐19 pandemic. Journal of Medical Ethics, 46(8), 510–513. 10.1136/medethics-2020-106398 32546658 PMC7316119

[jpm13099-bib-0011] Crocker, K. M. , Gnatt, I. , Haywood, D. , Butterfield, I. , Bhat, R. , Lalitha, A. R. N. , Jenkins, Z. M. , & Castle, D. J. (2022). The impact of covid ‐19 on the mental health workforce: A rapid review. International Journal of Mental Health Nursing, 32, 420–445. 10.1111/inm.13097 36461629 PMC9878253

[jpm13099-bib-0012] Doré, I. , O'Loughlin, J. , Sylvestre, M.‐P. , Sabiston, C. M. , Beauchamp, G. , Martineau, M. , & Fournier, L. (2020). Not flourishing mental health is associated with higher risks of anxiety and depressive symptoms in college students. Canadian Journal of Community Mental Health, 39(1), 34–48. 10.7870/cjcmh-2020-003

[jpm13099-bib-0013] Dyrbye, L. N. , Harper, W. , Moutier, C. , Durning, S. J. , Power, D. V. , Massie, F. S. , Eacker, A. , Thomas, M. R. , Satele, D. , Sloan, J. A. , & Shanafelt, T. D. (2012). A multi‐institutional study exploring the impact of positive mental health on medical Students' professionalism in an era of high burnout. Academic Medicine: Journal of the Association of American Medical Colleges, 87(8), 1024–1031. 10.1097/ACM.0b013e31825cfa35 22722352

[jpm13099-bib-0014] Elgohary, H. M. , Sehlo, M. G. , Bassiony, M. M. , Youssef, U. M. , Elrafey, D. S. , & Amin, S. I. (2021). Depression among health workers caring for patients with COVID‐19 in Egypt. The Egyptian Journal of Neurology, Psychiatry and Neurosurgery, 57(1), 139. 10.1186/s41983-021-00394-1 34690490 PMC8521320

[jpm13099-bib-0015] Fiorilli, C. (2015). Copenhagen burnout inventory (CBI): A validation study in an Italian teacher group. TPM: Testing, Psychometrics, Methodology in Applied Psychology, 22(4), 537–551. 10.4473/TPM22.4.7

[jpm13099-bib-0016] Ghio, L. , Patti, S. , Piccinini, G. , Modafferi, C. , Lusetti, E. , Mazzella, M. , & Del Sette, M. (2021). Anxiety, depression and risk of post‐traumatic stress disorder in health workers: The relationship with burnout during covid‐19 pandemic in Italy. International Journal of Environmental Research and Public Health, 18(18), 9929. 10.3390/ijerph18189929 34574851 PMC8469269

[jpm13099-bib-0058] Gonzalez, A. , Cervoni, C. , Lochner, M. , Marangio, J. , Stanley, C. , & Marriott, S. (2020). Supporting health care workers during the COVID‐19 pandemic: Mental health support initiatives and lessons learned from an academic medical center. Psychological Trauma: Theory, Research, Practice, and Policy, 12(S1), S168–S170. 10.1037/tra0000893 32584111

[jpm13099-bib-0017] Gray, M. , Monti, K. , Katz, C. , Klipstein, K. , & Lim, S. (2021). A “mental health PPE” model of proactive mental health support for frontline health care workers during the COVID‐19 pandemic. Psychiatry Research, 299, 113878. 10.1016/j.psychres.2021.113878 33756208 PMC7960034

[jpm13099-bib-0055] Huberty, C. J. , (2002). A History of Effect Size Indices. Educational and Psychological Measurement, 62(2), 227–240. 10.1177/0013164402062002002

[jpm13099-bib-0018] Hurst, K. T. , Ballard, E. D. , Anderson, G. E. , Greenstein, D. K. , Cavanaugh, G. W. , Dwyer, E. , Swartz, K. , Zarate, C. A. , Chung, J. Y. , & Park, L. T. (2022). The mental health impact of contact with COVID‐19 patients on healthcare workers in the United States. Psychiatry Research, 308, 114359. 10.1016/j.psychres.2021.114359 34995831 PMC8709733

[jpm13099-bib-0019] Iasiello, M. , van Agteren, J. , Keyes, C. L. M. , & Cochrane, E. M. (2019). Positive mental health as a predictor of recovery from mental illness. Journal of Affective Disorders, 251, 227–230. 10.1016/j.jad.2019.03.065 30927584 PMC6487880

[jpm13099-bib-0020] Keyes, C. L. M. (2002). The mental health continuum: From languishing to flourishing in life. Journal of Health and Social Behavior, 43(2), 207–222. 10.2307/3090197 12096700

[jpm13099-bib-0021] Keyes, C. L. M. (2005). Mental illness and/or mental health? Investigating axioms of the complete state model of health. Journal of Consulting and Clinical Psychology, 73(3), 539–548. 10.1037/0022-006X.73.3.539 15982151

[jpm13099-bib-0022] Keyes, C. L. M. (2006). Mental health in adolescence: Is America's youth flourishing? American Journal of Orthopsychiatry, 76(3), 395–402. 10.1037/0002-9432.76.3.395 16981819

[jpm13099-bib-0023] Keyes, C. L. M. , Dhingra, S. S. , & Simoes, E. J. (2010). Change in level of positive mental health as a predictor of future risk of mental illness. American Journal of Public Health, 100(12), 2366–2371. 10.2105/AJPH.2010.192245 20966364 PMC2978199

[jpm13099-bib-0024] Keyes, C. L. M. , Eisenberg, D. , Perry, G. S. , Dube, S. R. , Kroenke, K. , & Dhingra, S. S. (2012). The relationship of level of positive mental health with current mental disorders in predicting suicidal behavior and academic impairment in college students. Journal of American College Health, 60(2), 126–133. 10.1080/07448481.2011.608393 22316409

[jpm13099-bib-0025] Kristensen, T. S. , Borritz, M. , Villadsen, E. , & Christensen, K. B. (2005). The Copenhagen burnout inventory: A new tool for the assessment of burnout. Work & Stress, 19(3), 192–207. 10.1080/02678370500297720

[jpm13099-bib-0026] Labrague, L. J. , De los Santos, J. A. A. , & Fronda, D. C. (2021). Perceived COVID‐19‐associated discrimination, mental health and professional‐turnover intention among frontline clinical nurses: the mediating role of resilience. International Journal of Mental Health Nursing, 30(6), 1674–1683. 10.1111/inm.12920 34374480 PMC8447016

[jpm13099-bib-0027] Liang, L. , Yuan, T. , Guo, X. , Meng, C. , Lv, J. , Fei, J. , & Mei, S. (2022). The path of depression among frontline nurses during covid‐19 pandemic: A fuzzy‐set qualitative comparative analysis. International Journal of Mental Health Nursing, 31(5), 1239–1248. 10.1111/inm.13033 35727700 PMC9350022

[jpm13099-bib-0028] Lovibond, P. F. , & Lovibond, S. H. (1995). The structure of negative emotional states: Comparison of the Depression Anxiety Stress Scales (DASS) with the Beck depression and anxiety inventories. Behaviour Research and Therapy, 33(3), 335–343. 10.1016/0005-7967(94)00075-u 7726811

[jpm13099-bib-0059] Maunder, R. , Hunter, J. , Vincent, L. , Bennett, J. , Peladeau, N. , Leszcz, M. , Sadavoy, J. , Verhaeghe, L. M. , Steinberg, R. ,& Mazzulli, T. (2003). The immediate psychological and occupational impact of the 2003 SARS outbreak in a teaching hospital, 168(10), 1245–1251. PMC154178 12743065

[jpm13099-bib-0029] Maideen, A. A. , Idris, D. R. , Lupat, A. , Chung, Y. F. , Haji‐Badarudin, H. , Suhai, H. , Abdullah, H. N. , Omar, H. , Kisut, R. , Abdul Rahman, H. , & Abdul‐Mumin, K. H. (2022). Nurses' mental health and coping strategies throughout covid ‐19 outbreak: A nationwide qualitative study. International Journal of Mental Health Nursing, 31(5), 1213–1227. 10.1111/inm.13031 35714038 PMC9349883

[jpm13099-bib-0030] Mascayano, F. , van der Ven, E. , Moro, M. F. , Schilling, S. , Alarcón, S. , Al Barathie, J. , Alnasser, L. , Asaoka, H. , Ayinde, O. , Balalian, A. A. , Basagoitia, A. , Brittain, K. , Dohrenwend, B. , Durand‐Arias, S. , Eskin, M. , Fernández‐Jiménez, E. , Freytes Frey, M. I. , Giménez, L. , Gisle, L. , … HEROES group . (2022). The impact of the COVID‐19 pandemic on the mental health of healthcare workers: Study protocol for the COVID‐19 HEalth caRe wOrkErS (HEROES) study. Social Psychiatry and Psychiatric Epidemiology, 57(3), 633–645. 10.1007/s00127-021-02211-9 35064280 PMC8782684

[jpm13099-bib-0031] Mendez‐Pinto, I. , Antuña‐Casal, M. , & Mosteiro‐Diaz, M. P. (2023). Psychological disorders among Spanish nursing students three months after COVID‐19 lockdown: A cross‐sectional study. International Journal of Mental Health Nursing, 32, 479–489. 10.1111/inm.13086 36330581 PMC9877867

[jpm13099-bib-0032] Merino‐Godoy, M. Á. , Yot‐Domínguez, C. , Conde‐Jiménez, J. , Ramírez Martín, P. , & Lunar‐Valle, P. M. (2022). The influence of emotional burnout and resilience on the psychological distress of nursing students during the COVID‐19 pandemic. International Journal of Mental Health Nursing, 31(6), 1457–1466. 10.1111/inm.13046 35938942 PMC9538541

[jpm13099-bib-0033] Morgan, T. L. , McFadden, T. , Fortier, M. S. , Tomasone, J. R. , & Sweet, S. N. (2020). Positive mental health and burnout in first to fourth year medical students. Health Education Journal, 79(8), 948–962. 10.1177/0017896920944206

[jpm13099-bib-0034] Narita, Z. , Okubo, R. , Sasaki, Y. , Takeda, K. , Takao, M. , Komaki, H. , Oi, H. , Mizoue, T. , Miyama, T. , & Kim, Y. (2023). covid ‐19‐related discrimination, ptsd symptoms, and psychological distress in healthcare workers. International Journal of Mental Health Nursing, 32(1), 139–146. 10.1111/inm.13069 36176263 PMC9538840

[jpm13099-bib-0035] O'Higgins, M. , Rojas, L. A. , Echeverria, I. , Roselló‐Jiménez, L. , Benito, A. , & Haro, G. (2022). Burnout, psychopathology and purpose in life in healthcare workers during COVID‐19 pandemic. Frontiers in Public Health, 10, 926328. 10.3389/fpubh.2022.926328 36052010 PMC9425829

[jpm13099-bib-0036] Pang, Y. , Fang, H. , Li, L. , Chen, M. , Chen, Y. , & Chen, M. (2021). Predictive factors of anxiety and depression among nurses fighting coronavirus disease 2019 in China. International Journal of Mental Health Nursing, 30(2), 524–532. 10.1111/inm.12817 33491299 PMC8014285

[jpm13099-bib-0037] Petrillo, G. , Capone, V. , Caso, D. , & Keyes, C. L. M. (2015). The Mental Health Continuum–Short Form (MHC–SF) as a measure of well‐being in the Italian context. Social Indicators Research, 121(1), 291–312. 10.1007/s11205-014-0629-3

[jpm13099-bib-0056] Sampaio, F. M. C. , Sequeira, C. A. , da, C. , & Lluch Canut, M. T. (2015). Nursing psychotherapeutic interventions: a review of clinical studies. Journal of Clinical Nursing, 24(15–16), 2096–2105. Portico. 10.1111/jocn.12808 25819026

[jpm13099-bib-0039] Sani, G. , Janiri, D. , Moccia, L. , Albert, U. , Carrà, G. , Carmassi, C. , Cirulli, F. , Dell'Osso, B. , Menculini, G. , Nanni, M. G. , Pompili, M. , Volpe, U. , & Fiorillo, A. (2022). Psychopathological burden and coping strategies among frontline and second‐line Italian healthcare workers facing the COVID‐19 emergency: Findings from the COMET collaborative network. Journal of Affective Disorders, 311, 78–83. 10.1016/j.jad.2022.05.006 35533774 PMC9074380

[jpm13099-bib-0040] Schotanus‐Dijkstra, M. , Keyes, C. L. M. , de Graaf, R. , & ten Have, M. (2019). Recovery from mood and anxiety disorders: The influence of positive mental health. Journal of Affective Disorders, 252, 107–113. 10.1016/j.jad.2019.04.051 30981053

[jpm13099-bib-0041] Stab, N. , & Hacker, W. (2020). A pilot study on the possibility of human‐centred participative redesign of work organization at psychiatric wards. Journal of Psychiatric and Mental Health Nursing, 27(5), 497–508. 10.1111/jpm.12598 31957114

[jpm13099-bib-0042] Toh, W. L. , Meyer, D. , Phillipou, A. , Tan, E. J. , Van Rheenen, T. E. , Neill, E. , & Rossell, S. L. (2021). Mental health status of healthcare versus other essential workers in Australia amidst the COVID‐19 pandemic: Initial results from the collate project. Psychiatry Research, 298, 113822. 10.1016/j.psychres.2021.113822 33652251 PMC7902230

[jpm13099-bib-0043] Tomlin, J. , Dalgleish‐Warburton, B. , & Lamph, G. (2020). Psychosocial support for healthcare workers during the COVID‐19 pandemic. Frontiers in Psychology, 11, 1960. 10.3389/fpsyg.2020.01960 32849149 PMC7431467

[jpm13099-bib-0044] Vallée, A. (2023). Geoepidemiological perspective on COVID‐19 pandemic review, an insight into the global impact. Frontiers in Public Health, 11, 1242891. 10.3389/fpubh.2023.1242891 37927887 PMC10620809

[jpm13099-bib-0045] van Agteren, J. , Iasiello, M. , Lo, L. , Bartholomaeus, J. , Kopsaftis, Z. , Carey, M. , & Kyrios, M. (2021). A systematic review and meta‐analysis of psychological interventions to improve mental wellbeing. Nature Human Behaviour, 5(5), 631–652. 10.1038/s41562-021-01093-w 33875837

[jpm13099-bib-0046] Veitch, P. , & Richardson, K. (2021). Nurses need support during Covid‐19 pandemic. Journal of Psychiatric and Mental Health Nursing, 28(2), 303–304. 10.1111/jpm.12666 32558102 PMC7323110

[jpm13099-bib-0047] Vescovelli, F. , Cesetti, G. , & Ruini, C. (2020). Optimal well‐being, depression, and caregiving: An explorative investigation. Clinical Gerontologist, 43(5), 572–584. 10.1080/07317115.2019.1702130 31826711

[jpm13099-bib-0048] Wang, P. , Tang, Y. , Chen, Y. , He, Y. , Li, L. , Han, X. , Liu, Y. , Liu, T. , Liu, H. , Jiang, F. , & Zhu, J. (2023). Mental health status of mental health nurses in China: Results from a national survey. Journal of Psychiatric and Mental Health Nursing, 30(3), 547–557. 10.1111/jpm.12893 36577690

[jpm13099-bib-0049] Webb, L. (2021). The psychosocial risk of being “extremely vulnerable” during COVID‐19 and the role of behaviour activation. Journal of Psychiatric and Mental Health Nursing, 28(3), 505. 10.1111/jpm.12684 32888376

[jpm13099-bib-0050] Westerhof, G. J. , & Keyes, C. L. M. (2010). Mental illness and mental health: The two continua model across the lifespan. Journal of Adult Development, 17(2), 110–119. 10.1007/s10804-009-9082-y 20502508 PMC2866965

[jpm13099-bib-0051] Wilson, C. A. , Metwally, H. , Heavner, S. , Kennedy, A. B. , & Britt, T. W. (2022). Chronicling moral distress among healthcare providers during the COVID‐19 pandemic: A longitudinal analysis of mental health strain, burnout, and maladaptive coping behaviours. International Journal of Mental Health Nursing, 31(1), 111–127. 10.1111/inm.12942 34644443 PMC8653372

[jpm13099-bib-0052] Wood, A. M. , & Joseph, S. (2010). The absence of positive psychological (eudemonic) well‐being as a risk factor for depression: A ten year cohort study. Journal of Affective Disorders, 122(3), 213–217. 10.1016/j.jad.2009.06.032 19706357

[jpm13099-bib-0057] Zaçe, D. , Hoxhaj, I. , Orfino, A. , Viteritti, A. M. , Janiri, L. , & Di Pietro, M. L. (2021). Interventions to address mental health issues in healthcare workers during infectious disease outbreaks: A systematic review. Journal of Psychiatric Research, 136, 319–333. 10.1016/j.jpsychires.2021.02.019 33636688 PMC7880838

[jpm13099-bib-0053] Zhang, X. , Jiang, X. , Ni, P. , Li, H. , Li, C. , Zhou, Q. , Ou, Z. , Guo, Y. , & Cao, J. (2021). Association between resilience and burnout of front‐line nurses at the peak of the COVID‐19 pandemic: Positive and negative affect as mediators in Wuhan. International Journal of Mental Health Nursing, 30(4), 939–954. 10.1111/inm.12847 33893718 PMC8251287

